# The effects of endoscopic procedures and flatus tube application on early recurrence following endoscopic detorsion in sigmoid volvulus: Review of 792 patients

**DOI:** 10.12669/pjms.41.8.12514

**Published:** 2025-08

**Authors:** Esra Disci, Sabri Selcuk Atamanalp, Refik Selim Atamanalp, Cansu Tatar Atamanalp

**Affiliations:** 1 Esra Disci, MD Associate Professor, Department of General Surgery, Faculty of Medicine, Ataturk University, Erzurum, Turkiye; 2 Sabri Selcuk Atamanalp, MD Professor, Department of General Surgery, Faculty of Medicine, Ataturk University, Erzurum, Turkiye; 3 Refik Selim Atamanalp, MD Assistant Professor, Department of Medical Pathology, Prof. Dr. Cemil Tascioglu City Hospital, Istanbul, Turkiye; 4 Cansu Tatar Atamanalp, MD Assistant Professor, Department of Pediatrics, Haseki Education and Research Hospital, Istanbul, Turkiye

**Keywords:** Sigmoid volvulus, Endoscopic detorsion, Rigid endoscopy, Flexible endoscopy, Flatus tube

## Abstract

**Objectives:**

Endoscopic detorsion is the first therapeutic option with 80-90% success rates in uncomplicated sigmoid volvulus (SV). However, recurrence rate rises up to 75%, 13% of which occur in early period. In this study, we aimed to utilize the role of endoscopic procedures (rigid or flexible) and flatus tube application on early SV recurrence.

**Methodology:**

In Ataturk University, Faculty of Medicine, Research Hospital, Department of General Surgery, in a partial retrospective and prospective evaluation, early recurrence rates of 792 SV patients (72.8%) treated with endoscopic detorsion were evaluated.

**Results:**

In 58.5-year period, endoscopic detorsion was tried in 792 patients (rigid endoscopy in 351, while flexible endoscopy in 441) of total 1,088 SV cases. Among patients with successful endoscopic detorsion and viable bowel, early recurrence rates were 3.3% (9/274), 4.3% (11/254) and 18.1% (17/94) in rigid endoscopy with flatus tube application, flexible endoscopy with and without flatus tube application groups, respectively. Early recurrence rates were statistically similar between flatus tube-applied groups irrespectively of endoscopy technique (3.3% and 4.3%, in rigid and flexible endoscopy groups, respectively, P>0.05). However, early recurrence rate was significantly higher in flexible endoscopy without flatus tube application group when compared with that of rigid endoscopy and flexible endoscopy with flatus tube application groups (18.1% vs 3.3% and 18.1% vs 4.3%, respectively, P<0.001 in each).

**Conclusion:**

Early recurrence looks like independent from the kind of the endoscopic instruments in SV. Although flatus tubes may prevent early recurrence as long as their placement, early SV recurrence may occur in some patients following their removal.

## INTRODUCTION

Current management of uncomplicated sigmoid volvulus (SV) consists of endoscopic detorsion, which provides 80-90% of success rates.^1^ However, the disease is associated with a high recurrence rate up to 75% and approximately 13% of which occur in early period, during a few hours or days following detorsion.^2^,^3^ For this reason, some practitioners traditionally apply flatus tubes to obtain further detorsion and to prevent early SV recurrence.^4^ Although the utility of endoscopic procedures (rigid or flexible, sigmoidoscope or colonoscope) is well discussed in the literature, the role of the type of the endoscopes on the early SV recurrence is still controversial.^5^,^6^ Similarly, the relationship between flatus tube application and early SV recurrence is still an under-researched subject.^5^,^7^

In this study, to utilize the effects of endoscopic procedures (rigid or flexible) and flatus tube application on the early SV recurrence, we investigated the clinical outcomes of endoscopy-applied 792 patients (72.8%) among total 1,088 SV cases, who were treated during the last 58.5 years (from June 1966 to January 2025) in Ataturk University, Faculty of Medicine, Research Hospital, Department of General Surgery. The presented data actually signifies the most extensive single-center SV documentation over the world.^8^

## METHODOLOGY

### Study center:

This study was performed in Ataturk University, Faculty of Medicine, Research Hospital, Department of General Surgery.

### Patient Population:

Total 1,088 patients with SV and particularly 805 cases (74.0%), in whom non-operative detorsion was attempted, were evaluated.

### Evaluation Method:

Among total 1,088 SV patients, retrospective review was performed in 612 cases (56.3%), who were treated between June 1966 to July 1986. Additionally, 476 patients (43.8%), who were treated from June 1986 to January 2025, were evaluated prospectively.

### Inclusion-Exclusion Criteria:

Among 805 patients with non-operative detorsion attempt, all of endoscopically treated 792 patients (98.4%) were included the study, while 13 cases (1.6%) treated with barium enema were excluded.

### Premedication:

Propofol (2.5 mg/kg, intravenous) was required in a limited patient population.

### Instruments:

In rigid endoscopy, rigid sigmoidoscopes (2-cm diameter and 30 cm-length) were used. In flexible endoscopy, flexible sigmoidoscopes (1.2- to 1.5-cm diameter and 60- to 70-cm length) were frequently preferred, while flexible colonoscopes (1.2- to 1.5-cm diameter and 150- to 170-cm length) were occasionally used. As flatus tubes, silastic rectal tubes (1- to 1.5-cm diameter and 25- to 30-cm length) were generally preferred, while silastic sigmoid tubes (0.8- to 1.2-cm. diameter and 50- to 70-cm length) were rarely used.

### Endoscopy Technique:

Following the determination of the volvulus point at 20- to 30-cm distance from anal verge, the endoscopes were gently pushed towards the proximal and opposite side of the torsion accompanied by minimal air insufflation. When the endoscopes got past the obstructive line with gas or stool discharge and abdominal relaxation, the detorsion procedure was accepted as successful. If detorsion was not obtained in 15- to 20-minute period, it was accepted as unsuccessful, which required the termination of the procedure and emergency surgical treatment. Similarly, in cases with gangrenous mucosa, endoscopy was immediately terminated and such patients were treated with emergency surgery.

### Flatus Tube Application:

In patients with flatus tube application, the tubes were placed under endoscopic guidance. An adhesive bandage was used in the fixation of the tubes and they were removed following 24-hour period except for spontaneous discharge or removal by the patients or practitioners.

### Time-Practice Relationship:

Rigid endoscopy with flatus tube application were used from June 1966 to July 1988. Between July 1988 to January 2003, both rigid and flexible endoscopy with flatus tube application were preferred. Ever since, flexible endoscopy was always used with (from January 2003 to July 2011), with/without (between July 2011 to January 2023) and without (from that time) flatus tube application.

### Evaluation Criteria:

The presence of initial features of SV (abdominal pain/tenderness, distention and obstipation) in addition to radiological findings in abdominal X-ray radiography (coffee-bean sign) or computed tomography (mesenteric whirl sign) in the initial hospitalization period during the first few hours or days was accepted as early SV recurrence. Due to the absence of early recurrence in patients with flatus tube as long as tube placement, recurrence following the spontaneous discharge or removal of the tubes by the patients or practitioners was also accepted as early recurrence. In all cases with early recurrence, the data on endoscopic instrumentation (rigid or flexible) and flatus tube application (with or without) were noted.

### Statistical Evaluation:

IBM SPSS 20 program was used in statistical evaluation. Numbers and percentages were used in the presentation of the data and Pearson Chi-square test was used for comparisons between the numbers and percentages. Statistical significance level was set as p<0.05.

### Ethical Statement:

Ethical approval (Institutional Ethical Committee, 31 Jan 2025, B.30.2.ATA.0.01.00/03) and written informed statements were obtained.

## RESULTS

**Fig.1:**
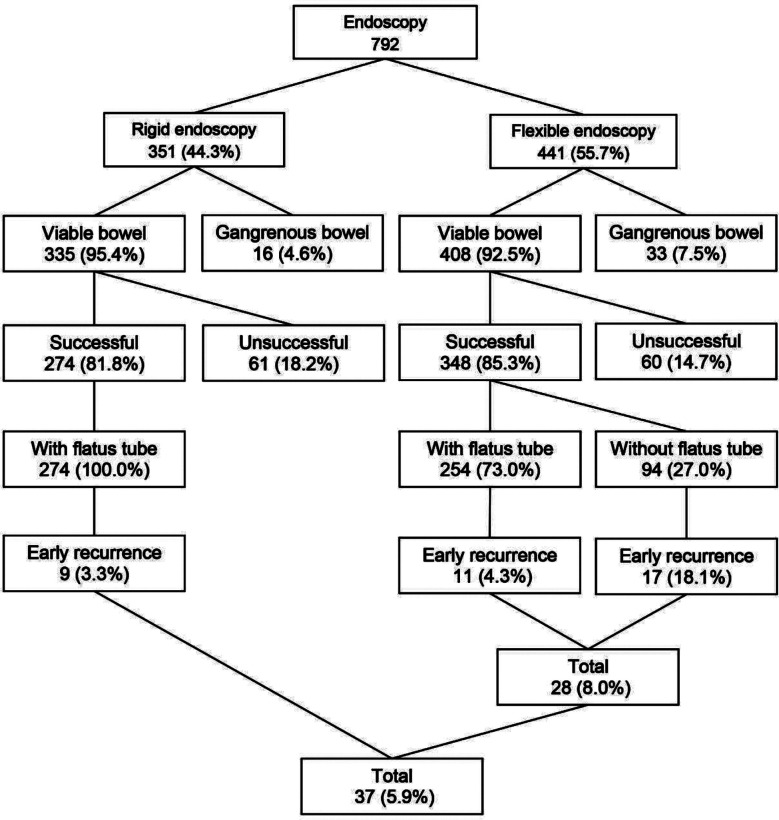
Endoscopic procedures, flatus tube applications and early recurrence rates in patients with sigmoid volvulus.

Endoscopic procedures and flatus tube applications in patients with SV are summarized in Fig.1, while detailed results and related statistical analyses are given in Table-I. In 58.5-year period, endoscopic detorsion was tried in 792 patients (72.8%) of total 1,088 SV cases. Rigid endoscopic detorsion was tried in 351 patients (44.3%) with 81.8% success rate (274 cases) among 335 patients (95.4%) with viable bowel. On the contrary, flexible procedure was applied in 441 cases (55.7%) with 85.3% success rate (348 patients) among 408 cases (92.5) with viable bowel.

**Table-I:** Early recurrence rates according to the used endoscopic procedures and flatus tube application in patients with sigmoid volvulus.

Parameter	n	Recurrence	Statistical analysis
Rigid endoscopy with flatus tube application	274	9 (3.3%)^a^	***Chi-square test*** X^2^=0.396; P=0.529 (a-b) X^2^=23.349; P<0.001 (a-c) X^2^=17.543; P<0.001 (b-c)

In this series, total early recurrence rate was 5.9% (37 of 622 cases with successful endoscopic detorsion). Although there was no recurrence as long as tube placement, following the spontaneous discharge or removal of the tubes, early recurrence rate was 3.3% (9/274) in rigid endoscopy with flatus tube application group and 4.3% (11/254) in flexible endoscopy with flatus tube application group. On the other hand, this rate was 18.1% (17/94) in flexible endoscopy without flatus tube application and 8.0% (28/348) in total flexible endoscopy groups. Statistical analysis demonstrated that, early recurrence rates were statistically similar in flatus tube-applied patients irrespectively of endoscopy technique (3.3% and 4.3%, in rigid and flexible endoscopy groups, respectively, P>0.05). However, early recurrence rate was statistically higher in flexible endoscopy without flatus tube application group when compared with that of rigid endoscopy and flexible endoscopy with flatus tube application groups (18.1% vs 3.3% and 18.1% vs 4.3%, respectively, P<0.001 in each).

## DISCUSSION

In the management of uncomplicated SV, endoscopic detorsion is the current option even in pregnant women and children.^9^-^12^ However, due to presence of dolichosigmoid (an elongated and dilated sigmoid colon with an elongated mesentery, an anatomical predisposition to SV) in most cases, recurrence is common with a mean rate of 75% (range: 3%-86%) and almost 13% of which occur in the first few hours or days as ‘early recurrence’.^2^-^4^,^6^,^13^,^14^

In the present series, early recurrence rate was 5.9%. Our results demonstrated no correlation between the type of the used endoscopes (rigid or flexible) and early recurrence, while the sole effective factor in the development of early recurrence was determined as the usage of flatus tubes. These results raised the topics of the types, usage and removal of the flatus tubes following endoscopic detorsion in SV.

Although advanced age, early onset, male gender, high-fiber diet habit, living in high altitude and chronic constipation are possible risk factors affecting SV recurrence, their role on early recurrence is controversial.^15^-^18^ Similarly, the success and complication rates as well as the user friendliness and patient adoption of rigid and flexible endoscopes (sigmoidoscopes or colonoscopes) are widely discussed in the literature. However, their role on SV recurrence, particularly on early recurrence is an undiscussed subject.^6^,^19^,^20^ Regarding the relationship between flatus tubes and early recurrence, no recurrent case was reported during the usage of the tubes in relatively large series.^6^,^21^-^25^ Our data supported this finding. However, some flatus tubes spontaneously displace due to degassification or defecation during the clinical observation period following endoscopic detorsion. Additionally, due to the pain and discomfort, some patients or practitioners remove them. In such circumstances, early recurrence may occur following the discharge of the tubes irrespectively of their types (short or long, narrow or large).^6^

The relationship between SV and its recurrence is not a mystery and most details of causes, pathogenesis, clinical presentation, diagnosis, and treatment of this clinical entity are discussed in detail. However, early recurrence is a relatively uncommon clinical picture in SV.^5^ For this reason, our data on causes and prevention of early SV recurrence is an important improvement. It is clear that, new wide-spectrum studies utilizing our findings as well as other details of early SV recurrence will illuminate the clinical evaluation of this relatively rare but serious entity.

In conclusion, based on our results, we may discuss that early SV recurrence looks like independent from the kind of the endoscopic instruments (rigid or flexible). Conversely, flatus tubes may prevent early recurrence during their operating time. However, following their removal, early SV recurrence is a probable outcome in some patients.

### Limitations:

We used partial retrospective data, nonrandomized patient distribution and non-matched statistical evaluation, which were the limitations of the present study. However, prospective data, randomized sampling and matched statistical evaluation require tens of patients during a few decades in SV.

## CONCLUSIONS

Depending on the outcomes of our partial retrospective and prospective non-matched evaluation consisting of 27 patients with early SV recurrence among endoscopically treated 792 patients with SV, we determined early SV recurrence independent from the kind of the endoscopic instruments, while the usage of the flatus tubes reduced early SV recurrence. Prospective randomized clinical studies or matched analyses may shed some light on the novel approach.
